# Ants are the major agents of resource removal from tropical rainforests

**DOI:** 10.1111/1365-2656.12728

**Published:** 2017-08-08

**Authors:** Hannah M. Griffiths, Louise A. Ashton, Alice E. Walker, Fevziye Hasan, Theodore A. Evans, Paul Eggleton, Catherine L. Parr

**Affiliations:** ^1^ School of Environmental Sciences University of Liverpool Liverpool UK; ^2^ Department of Life Sciences Natural History Museum London UK; ^3^ School of Animal Biology The University of Western Australia Perth WA Australia; ^4^ School of Animal, Plant and Environmental Sciences University of the Witwatersrand Wits South Africa; ^5^ Department of Zoology & Entomology University of Pretoria Pretoria South Africa

**Keywords:** ecosystem function, ecosystem process, forager, functional redundancy, invertebrate, nutrient distribution, scavenger, soil

## Abstract

1. Ants are diverse and abundant, especially in tropical ecosystems. They are often cited as the agents of key ecological processes, but their precise contributions compared with other organisms have rarely been quantified. Through the removal of food resources from the forest floor and subsequent transport to nests, ants play an important role in the redistribution of nutrients in rainforests. This is an essential ecosystem process and a key energetic link between higher trophic levels, decomposers and primary producers.
2. We used the removal of carbohydrate, protein and seed baits as a proxy to quantify the contribution that ants, other invertebrates and vertebrates make to the redistribution of nutrients around the forest floor, and determined to what extent there is functional redundancy across ants, other invertebrate and vertebrate groups.
3. Using a large‐scale, field‐based manipulation experiment, we suppressed ants from *c*. 1 ha plots in a lowland tropical rainforest in Sabah, Malaysia. Using a combination of treatment and control plots, and cages to exclude vertebrates, we made food resources available to: (i) the whole foraging community, (ii) only invertebrates and (iii) only non‐ant invertebrates. This allowed us to partition bait removal into that taken by vertebrates, non‐ant invertebrates and ants. Additionally, we examined how the non‐ant invertebrate community responded to ant exclusion.
4. When the whole foraging community had access to food resources, we found that ants were responsible for 52% of total bait removal whilst vertebrates and non‐ant invertebrates removed the remaining 48%. Where vertebrates were excluded, ants carried out 61% of invertebrate‐mediated bait removal, with all other invertebrates removing the remaining 39%. Vertebrates were responsible for just 24% of bait removal and invertebrates (including ants) collectively removed the remaining 76%. There was no compensation in bait removal rate when ants and vertebrates were excluded, indicating low functional redundancy between these groups.
5. This study is the first to quantify the contribution of ants to the removal of food resources from rainforest floors and thus nutrient redistribution. We demonstrate that ants are functionally unique in this role because no other organisms compensated to maintain bait removal rate in their absence. As such, we strengthen a growing body of evidence establishing ants as ecosystem engineers, and provide new insights into the role of ants in maintaining key ecosystem processes. In this way, we further our basic understanding of the functioning of tropical rainforest ecosystems.

## Graphical abstract

In tropical rainforest, ants are ecologically dominant, and are up to 25% of the total animal biomass; however, the contribution of ants compared with other organisms to ecosystem processes has yet to be quantified. The authors show that ants are essential in redistributing nutrients around in ecosystems, and this function cannot be compensated for by other groups.

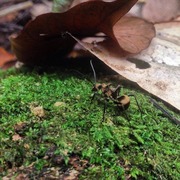

## 1 INTRODUCTION

Tropical forests are globally important ecosystems. They hold more than half the Earth's terrestrial species (Dirzo & Raven, 2003) and store huge amounts of carbon (Berenguer et al., 2014; Cramer et al., 2004). Within tropical forests, ants (Hymenoptera: Formicidae) are a dominant invertebrate group (Lach, Parr, & Abbott, 2010), estimated to make up to 25% of animal biomass (Hölldobler & Wilson, 1990), and are recognised as ecosystem engineers (Folgarait, 1998). Recent work has demonstrated that diversity in ants and other invertebrate groups is positively associated with ecosystem functioning in rainforest systems (Fayle et al., 2011; Griffiths et al., 2015). However, little is known about the relative contribution of ants to ecosystem processes compared with other functionally similar groups, or the capacity of organisms to compensate to maintain processes in the event of anthropogenic driven changes in biotic communities.

Because of their dominance and abundance within tropical forests, ants are widely cited as major contributors to the maintenance of ecological processes (e.g. Grimaldi & Engel, 2005; Hölldobler & Wilson, 1990). One such process is the redistribution of non‐plant derived organic material, including dead animal bodies, across forest floors (Fayle et al., 2011). Ants display a wide variety of feeding strategies; the majority of species are omnivorous scavengers, consuming plant, fungal and animal tissue, some are specialist predators of other invertebrates, whereas others feed on seeds, honeydew, plant nectar and fungi (Hölldobler & Wilson, 1990; Lach et al., 2010). Through the collection and transport of material, rich in nitrogen (N) and phosphorus (P), we know that ants carry out important roles in the redistribution and concentration of nutrients around ecosystems (Frouz & Jilková, 2008). However, we do not know the contribution ants make to the functioning of these systems, over large, ecologically meaningful scales. This is because until now investigations have used small‐scale experimental manipulations (e.g. Klimes, Janda, Ibalim, Kua, & Novotny, 2011; Wardle, Hyodo, Bardgett, Yeates, & Nilsson, 2011), or have been qualitative, based on descriptive and/or observational data (but see Parr, Eggleton, Davies, Evans, & Holdsworth, 2016).

Recent work has demonstrated that compared with vertebrates, invertebrates are the key agents of seed predation in old growth rainforests (Ewers et al., 2015). However, we know of no investigation that quantifies the specific contribution that ants make to seed removal or to the redistribution of food resources, and thus nutrients, within tropical forests, when compared with other non‐ant invertebrates or vertebrates. It is important that we address this knowledge gap because understanding the extent to which organisms carry out functionally similar roles in an environment provides information on the resilience of that ecosystem to species losses (e.g. Houadria et al., 2016; Laliberté et al., 2010). The redundancy hypothesis (Grime, 1997) proposes that loss of species will not affect ecosystem processes as long as there are functionally similar species that act as ecological insurance (Yachi & Loreau, 1999), compensating for ecosystem functioning in their absence. Here, we expand this hypothesis to encompass not just the ability of different species within the same taxa to carry out similar functions, but propose there may also be redundancy between different taxonomic groups. For example, it is possible that the scavenging and subsequent nutrient distribution role of ants in rainforests could be carried out by other invertebrates and vertebrates, which would indicate a resilience of this function to changes in the structure of animal communities. However, we currently lack the empirical evidence to address this issue, meaning we do not know how anthropogenic driven shifts in biotic communities are likely to influence the maintenance of ecosystem functioning in rainforests, which are rapidly changing ecosystems (Barlow et al., 2016; Hansen et al., 2008).

We quantified the contribution of ants, other invertebrates and vertebrates to the removal of food resources and thus the redistribution of nutrients within a tropical rainforest, to assess the roles that the different groups play in ecosystem function, and the capacity for functional redundancy within and between these groups. We investigated this with a large‐scale manipulative field experiment in an old growth tropical rainforest in Malaysian Borneo. We used the removal of food baits as a proxy for the redistribution of nutrients within ant suppression and control plots. A combination of caged and open treatments meant food resources were available to either the whole foraging community (all invertebrates and vertebrates) or invertebrates only in control plots, whereas food resources were available to either vertebrates plus non‐ant invertebrates, or to non‐ant invertebrates only in the ant suppression plots. This design allowed us to address three questions: (i) What is the relative contribution of ants, non‐ant invertebrates and vertebrates to nutrient redistribution around the forest floor? (ii) Are non‐ant organisms able to compensate to maintain the same level of bait removal when ants are suppressed? (iii) With ant suppression, is there a change in non‐ant invertebrate abundance and composition at baits? Our experimental framework allowed us to partition the contribution of each group to the removal of food resources and thus nutrient distribution. Additionally, we monitored the activity of major invertebrate groups over the 2‐year duration of the experiment.

Given the dominance of ants within rainforest systems, we predicted that: (i) Ants are the major agents of nutrient redistribution, carrying out more of the process than any other group; (ii) In accordance with Parr et al. (2016), release from predation and interference competition would result in an increase in the abundance of non‐ant invertebrates with ant suppression; (iii) Although the abundance of non‐ant invertebrates will increase, the role of ants will not be compensated for functionally, meaning that where they are suppressed, there will be a significant decline in bait removal.

## 2 MATERIALS AND METHODS

### 2.1 Field site and ant suppressions

This study was carried out within an area of lowland, old growth dipterocarp rainforest in the Maliau Basin Conservation Area, Sabah, Malaysia (4°44′35″ to 55″ N and 116°58′10″ to 30″ E; mean annual rainfall 2,838 ± 93 mm). In October 2014, we established eight experimental plots within a 42‐ha area, each measuring 50 × 50 m, with an additional buffer zone of 15 m surrounding treatment plots; sampling was confined to the central 50 × 50 m on treatment plots. Four plots were allocated as control and four as ant suppression plots, each separated by at least 100 m. We applied two poison bait types to the ant suppression plots: Synergy Pro^®^ (active ingredients: hydramethylnon and pyriproxyfen) and a custom bait, which consisted of Whiskas^®^ cat food soaked in a sugar solution (60 g/L sugar in water) containing imidacloprid at a concentration of 110 p.p.m. The combination of these two poison bait types was used to ensure ants with different food preferences were attracted to the baits. Suppression of ants began in October 2014 through an initial application of 7.1 kg/ha Synergy Pro^®^ and 8 kg/ha custom bait, which were scattered equally by hand across the entire 80 × 80 m area of each plot. In subsequent poison applications, Synergy Pro^®^ was applied at 2.5 kg/ha to the central 50 × 50 m sampling area and buffer zone and the custom bait was applied at 4.1 kg/ha to the buffer zone only. To maintain the ant suppression treatment, while avoiding the application of excessive amounts of insecticides, we applied an integrated pest management approach. If ant activity was greater than or equal to 20% of that on the control plots, we reapplied baits. Using this novel, large‐scale ecosystem manipulation, we successfully suppressed the abundance of ants arriving at bait cards by an average of 90% (Appendix S1) and reduced ant abundance in the leaf litter by 87% (assessed using Winkler bag extractions in 2014 and 2015).

This baiting approach was similar to that used by Parr et al. (2016) in that it was specifically designed to minimise detrimental effects on non‐target organisms in the following ways: (i) the poison baits have low toxicity to terrestrial vertebrates and plants (Etigra, 2006; Sumitomo Chemical, 2016); (ii) the size and composition of the baits are designed to appeal to ants, and while they may appeal to some small mammals, we applied the baits during the day when ants are at their most active and these organisms are less active; (iii) once collected and returned to the nest, these baits are unavailable to surface‐foraging organisms; (iv) the quantities applied to suppression plots were below biologically relevant levels. For example, the amount of insecticides in the foraging territories of even the smallest, most vulnerable mammals, such as shrews and mice, was lower than the LD50s. Therefore, the amount of insecticide applied would be insufficient to kill small vertebrates, even if they were able to find and eat all of the bait spread over their foraging territories before it was removed by ants. Finally, data from Winker bag extractions demonstrate that the abundance of non‐ant invertebrates on the ant suppression plots was either equal to or significantly higher compared with control plots (P. Eggleton, unpublished data).

Ant and non‐ant invertebrate activity were assessed every 2 weeks using monitoring baits. On two 50‐m transects in the centre of the plots, we placed 0.3 g Whiskas^®^ cat food onto twenty 5 × 5 cm laminated cards, each separated by 5 m. These were left undisturbed for 1 hr, after which they were checked and the numbers of ants and non‐ant invertebrates were recorded. It was not possible to accurately count the exact numbers of ants in the field, so instead, following Parr et al. (2016), we estimated numbers using a ranked 1–6 scale (0 = 0 ants; 1 = 1 ant; 2 = 2–5 ants; 3 = 6–10 ants; 4 = 11–20 ants; 5 = 21–50 ants; 6 = >50 ants). Non‐ant invertebrates were visually categorised into major group and abundance was recorded: wasp (Hymenoptera), cricket (Orthoptera), fly (Diptera), springtail (Collembola), beetle (Coleoptera), cockroach (Blattodea), spider (Araneae) and harvestman (Opiliones).

### 2.2 Resource removal experiments

During September and October 2016, we established 30 bait removal stations (15 open and 15 caged) within the core 50 × 50 m sampling area of each experimental plot. At each station, food resources were placed in an open Petri dish (6 cm width; 1.5 cm depth) either directly onto the forest floor (open treatment) or within a 20 × 20 × 20 cm metal mesh cage (caged treatment: Appendix S2 for photograph examples of caged and open treatments). The mesh‐size (1 × 1 cm) of the cages ensured no vertebrates could access the baits within the caged treatment, but did not inhibit the access of the majority of invertebrates. Three bait types were used: 3.05 g (± 0.02 g) of dried carbohydrate bait (biscuit); 3.04 g (± 0.02 g) of dried seed bait (sunflower seed); and 1.08 g (± 0.01 g) of dried protein bait (fish; a smaller amount of protein bait was used because it was less dense and thus occupied a larger volume than the other bait types). See Appendix S2 for more details of baits used. The bait types were selected to mimic the foraging resources available in the natural system such as sugar‐rich fruits and nectar, seeds and dead animal bodies and attract as wide a diversity of foraging organisms as possible. We therefore used food resources that were carbohydrate, protein or seed, and importantly, selected resources that we could easily measure the amount removed. Using bait assays in this way is a standard approach in ant ecology (e.g. Fayle et al., 2011; Houadria et al., 2016; Kaspari, Donoso, Lucas, Zumbusch, & Kay, 2012). Baits were dried at 50°C for 2 days to a constant mass (assessed using a Ohaus^™^ balance, 0.01 g precision) before placement in the field. Resource removal stations were separated by 5 m and each bait type (carbohydrate/seed/biscuit) × treatment (caged/open) was randomly placed on three 50‐m transects. Each transect was separated by 10 m. In each plot, bait type was replicated five times per treatment (total baits, *n* = 30 per plot) and this was repeated temporally on two different days (total *n* = 60 per plot, total *n* = 480; Appendix S2 for example of the plot layout). Both caged and open treatments were put onto the forest floor between 9 a.m. and 11 a.m. and protected from the rain by a plastic cover. After 24 hr, all baits were collected, transported to the laboratory, dried again at 50°C to constant mass and weighed.

### 2.3 Statistical analyses

All analyses were carried out using R version 3.2.3 (R Core Team, 2015). We used generalised mixed‐effects models (glmer) in the lme4 package (Bates, Mächler, Bolker, & Walker, 2015) to assess if plot treatment (ant suppression/control), cage treatment (caged/open) and bait type (carbohydrate/seed/protein) or the interaction between these factors influenced the amount of bait removed from each of the stations. In this model, the proportion of dry mass that remained after 24 hr in the field was the dependent variable and plot was included as a random factor. Because we used proportion data as our response, a binomial error distribution was specified with a logit‐link function (e.g. model ← glmer(prop.gone ~ plot.treatment*cage.treatment*bait + (1|plot), family = binomial(link = “logit”), data = bait). Bearded pigs (*Sus barbatus*) destroyed a total of 103 bait stations; these were removed from analyses (the likelihood of a station being attacked by pigs was not significantly affected by plot treatment, cage treatment or bait type: Appendix S3).

To investigate if the ant suppression treatment influenced the abundance of non‐ant invertebrates recorded at the bait monitoring cards, pooled abundances of each non‐ant invertebrate major group were tested in separate models. Treatment was included as a fixed effect; sampling period and plot were included as separate random factors. This approach was to account for lack of temporal independence arising from different plots being sampled within the same time period, and for lack of spatial independence as a result of repeatedly sampling on the same plot. Models were over‐dispersed, and as such, we used a negative‐binomal glmer (using the function glmer.nb: e.g. m.fly ← glmer.nb(fly ~ treatment + (1|plot) + (1|day), data = invert). Finally, we performed a multivariate analysis of variance (adonis test) within the ‘vegan’ package (Oksanen et al., 2015) to assess if treatment influenced the community composition of the non‐ant invertebrates.

A top‐down approach was used to arrive at the best descriptive model (Zuur, Ieno, Walker, Saveliev, & Smith, 2009): all fixed effects and interactions were sequentially removed until a reduced minimum model was obtained, including only significant terms with *p* < .05. Chi‐squared likelihood ratio tests were used to assess the loss of explanatory power following the removal of an interaction or single‐term predictor.

## 3 RESULTS

**Figure 1:**
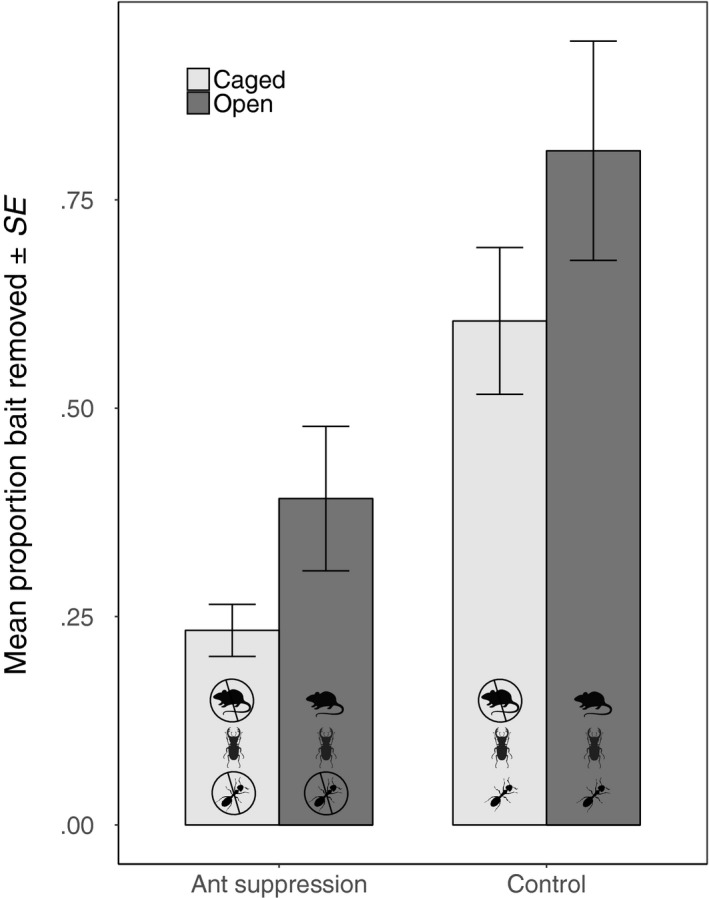
The mean proportion (±*SE*) of food resources removed from bait stations that were either caged (light grey bars: vertebrate exclusion) or open (dark grey bars: open to all foragers: invertebrates and vertebrates) within ant suppression and control plots

Plot treatment (ant suppression/control) ($\chi_{1}^{2}$ = 17.5, *p* < .001), cage treatment (caged/open) ($\chi_{1}^{2}$ = 14.8, *p* < .001) and bait type (carbohydrate/protein/seed) ($\chi_{1}^{2}$ = 25.8, *p* < .001), all significantly influenced the proportion of bait that was removed from the forest floor. Less bait was removed from the ant suppression plots compared with the control plots; less was removed from the caged stations compared with the open stations (Figure 1); and fewer seeds were removed compared with the carbohydrate and protein baits (Appendix S4). There were no significant interactions. Bait mass of open treatments (food resources available to all foragers) within the control plots declined by 80.0% (±*SE* = 9.2%) compared with a 59.5% (±*SE* = 10.9%) decline in the caged treatments (resources available to invertebrates only) in the control plots (Figure 1). This difference suggests that vertebrates remove an average of 25.6% of foraging resources and invertebrates remove the remaining 74.4%. Comparing the open treatments on the ant suppression and control plots enabled us to quantify the contribution of ants to bait removal compared with other non‐ant invertebrates and vertebrates combined. We found a decline in bait mass of 80.0% (±*SE* = 9.2%) within the open baits in the control plots compared with a 38.1% (±*SE* = 11.7%) decline in open baits the ant suppression plots (Figure 1); furthermore, this decline was consistently the case for all bait types (Appendix S4). Therefore, ants were responsible for 52% of bait removal compared with all other organisms (invertebrates and vertebrates). Finally, in terms of the contribution of ants to invertebrate‐mediated bait removal (i.e. considering the caged stations only): bait mass declined by 59.5% (±*SE* = 10.9%) in the control compared with a decline of 23.1% (±*SE* = 9.5%) in the ant plots (Figure 1). This difference suggests that at least 61% of invertebrate‐mediated scavenging is carried out by ants and the remaining 39% by all other invertebrates.

**Figure 2:**
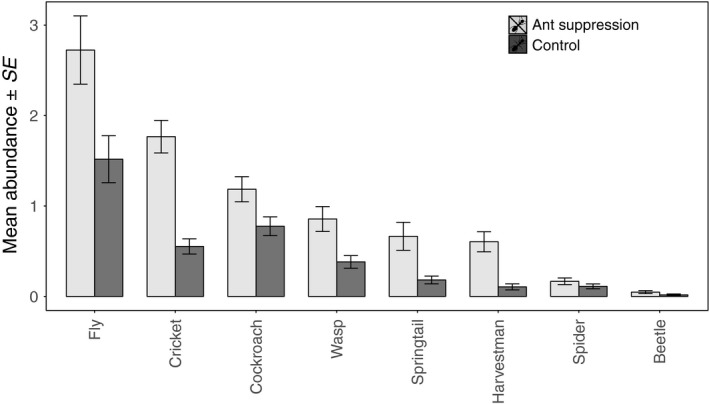
The mean abundance (±*SE*) of non‐ant invertebrates observed at monitoring baits in ant suppression (light grey bars) and control plots (dark grey bars)

**Figure 3:**
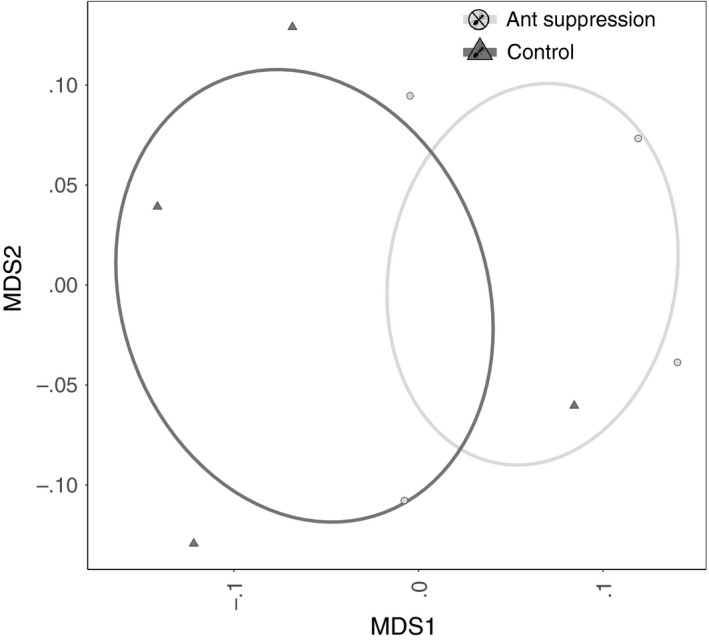
A nonmetric multidimensional scaling (NMDS) ordination of the non‐ant invertebrate communities within ant suppression (light grey circles) and control plots (dark grey triangles)

Treatment significantly affected the abundance of all non‐ant invertebrate groups, except beetles and spiders, observed at the monitoring baits (Table 1). In all cases, there were more individuals observed in the ant suppression plots compared with the control plots (Figure 2): the abundance of flies, crickets, wasps springtails and harvestmen recorded at the monitoring baits in the ant suppression plots was 80% higher than observed in the control plots, while the abundance of cockroaches increased by around 50%. Consequently, there was a significant shift in the composition of the invertebrate community recorded in the control, compared with the treatment plots (*F*_1,6_ = 12, *p* = .03; Figure 3).

**Table 1:** Model outputs of negative binomial generalised linear mixed‐effects models to assess the impact of ant suppression on the abundance of non‐ant invertebrates in experimental plots. Groups that were significantly affected (*p* ≥ .05) are highlighted in bold, those that were not significantly affected are italicized, significance was determined using a likelihood ratio test (LRT)

Group	LRT	*df*	*p*
Fly	**14.03**	**1**	**<.0001**
Cricket	**13.00**	**1**	**<.0001**
Cockroach	**4.36**	**1**	**.037**
Wasp	**5.93**	**1**	**.015**
Springtail	**6.57**	**1**	**.01**
Harvestman	**5.06**	**1**	**.024**
Spider	*1.33*	1	*.248*
Beetle	*0.94*	1	*.333*

## 4 DISCUSSION

In this study, we used a novel field manipulation experiment to quantify the relative contribution of ants to a key tropical forest ecosystem process. In doing so, we have demonstrated for the first time, what has been long predicted, that ants are the major agents of resource removal in these systems. Many papers reference ants as the most functionally important invertebrate group in tropical systems—one of the “the little things that run the world” (Wilson, 1987). However, until now these assertions had not been verified by empirical evidence in tropical forests. Previous work on ant‐mediated ecosystem functioning has tended to focus on seed dispersal (Gove, Majer, & Dunn, 2007), bioturbation of soil (Folgarait, 1998) or symbiotic food web interactions (Currie, 2001; Parr et al., 2016). Here, we have quantified the role of ants in scavenging and thus nutrient redistribution, which is an essential and often overlooked aspect of decomposition, linking higher trophic level organisms, decomposers and plants (Frouz & Jilková, 2008). As such, we provide new insights into the role of ants in maintaining key ecosystem processes and further our understanding of the functioning of tropical rainforest ecosystems.

Our estimates suggest that ants are responsible for a minimum of 52% of bait removal when compared with all other groups (vertebrates and non‐ant invertebrates), and for 61% of invertebrate‐mediated scavenging. Although ants display a large range of feeding strategies, most forage for small, widely dispersed food, including dead vertebrates, invertebrates, seeds and animal waste, which are then taken to nests (Carroll & Janzen, 1973). This collection and transport of material, rich in nitrogen (N) and phosphorus (P), results in the redistribution and concentration of nutrients around ecosystems, influencing soil biota and vegetation (Frouz & Jilková, 2008). For example, Wagner, Brown, and Gordon (1997) demonstrated that concentrations of key plant‐limiting nutrients and densities of micro‐arthropods and protozoa were significantly higher in ant nest soils, while ant mounds have been associated with increased seed production (Wagner, 1997). These studies were carried out in arid grasslands, not rainforests, so caution must be taken in making inferences between the systems. Nevertheless, these studies demonstrate the multi‐trophic impact that ant‐mediated nutrient redistribution can have on soils and vegetation. Small‐scale variation in soil nutrients and heterogeneity has been demonstrated to affect tropical forest diversity and plant community structure (John et al., 2007; Xu et al., 2016). Therefore, ant‐mediated nutrient redistribution is likely to be a key process in these systems with implications for forest composition and function. However, we are aware of no study to date that has focussed on the soil properties associated with ant nests in rainforest systems and as such we highlight this as an area in need of further investigation.

When only invertebrates had access to foraging resources, ants were responsible for 61% of bait removal, meaning that all other invertebrates combined removed the remaining 39% of baits. However, these figures are likely to be conservative estimates for two reasons. First, our suppression treatment was effective at reducing ant activity by an average of 90%. This means that around 10% of ants may have been actively contributing to bait removal on the treatment plots. Second, we observed a significant increase in non‐ant invertebrates arriving at monitoring cards in the ant suppression plots (explored further below). Therefore, other invertebrates were contributing more to the removal of baits on the ant suppression plots than would be observed under normal circumstances (i.e. with no ant suppression). It is likely then that this study underestimates the true contribution ants make to the movement of food resources within tropical forests. Nevertheless, our study highlights the fundamental contribution that ants make to the removal of foraging resources from tropical forest floors, thus illustrating their key role in soil nutrient cycling and tropical forest function.

We showed that invertebrates are responsible for about three‐quarters of the removal of food resources from the forest floor, while vertebrates only accounted for around a quarter. Although it is possible that this result is partly driven by monitoring activities on experimental plots disturbing vertebrate communities, these figures are in line with work by Ewers et al. (2015), who reported that invertebrates removed 72% of seeds from old growth forest floors. We have built on these findings by demonstrating that this pattern holds true not only for seeds but also for other food resources; providing evidence of the importance of invertebrates for the cycling of both animal‐ and plant‐derived products in rainforests. Ewers et al. (2015) asserted that the functional importance of invertebrates was reduced in secondary forest, because mammals compensate and carry out many of the functional roles that are dominated by invertebrates in primary rainforests. However, it is unlikely that the removal of food resources by mammals can truly replace the ecological processes carried out by invertebrates, in particular ants. This is because ants concentrate nutrients in nests (Bestelmeyer & Wiens, 2003; Frouz & Jilková, 2008), leading to greater ecosystem heterogeneity and to hotspots of diversity (da SL Sternberg et al., 2007; Laakso & Setälä, 1998; Wagner et al., 1997). We cannot assume, therefore, that because two groups appear to carry out similar processes, they have identical effects on ecosystem function. The inter‐phylum redundancy reported by Ewers et al. (2015) may not actually mitigate the negative consequences of anthropogenic habitat disturbance. Instead, in very disturbed habitats where ant diversity has declined (e.g. Luke, Fayle, Eggleton, Turner, & Davies, 2014), we may see a homogenisation of diversity (c.f. de Castro Solar et al., 2015).

Ant suppression resulted in a shift in the abundance and composition of non‐ant invertebrates at monitoring baits, with a significant increase in the numbers of more than 50% of all groups except spiders and beetles. Thus, while these groups may have removed more baits than in the presence of ants, we found no evidence that this resulted in compensation in scavenging rates by these other groups. Invertebrates removed an average of 23% of baits from the caged stations in the ant suppression plots, while 60% of bait was removed from the equivalent stations within the control plots. Ants are opportunists and have been shown to find and remove food resources rapidly before other groups arrive (Fellers & Fellers, 1982; Wilson, 1987). Our results show that when ants are removed, the rate of discovery and removal of baits declines and is not compensated to any great extent by other groups. If complete compensation had occurred through the activity of other invertebrates, we would not expect there to be a significant difference in removal rate between the caged bait stations in the ant suppression plots and control plots. Therefore, we have demonstrated that it is not simply a matter of ants acting as the fastest and most efficient scavengers, but that they are likely to be functionally non‐replaceable in their foraging roles in rainforests. This finding is important because ant diversity is sensitive to habitat disturbances such as repeated logging or conversion to oil palm (Fayle et al., 2010; Klimes et al., 2012; Luke et al., 2014) and Fayle et al. (2011) found that ant species richness was directly related to the rate of food resource removal across a land‐use gradient. As anthropogenic habitat disturbances intensify to a point where ant diversity and abundance declines, the ant‐mediated ecosystem processes of scavenging and nutrient redistribution are also likely to decline, with uncertain knock‐on effects for other aspects of ecosystem functioning.

## AUTHORS' CONTRIBUTIONS

C.P., P.E., T.E., H.G. and L.A. conceived and designed the experiment; A.W., F.H., H.G. and L.A. collected the data; H.G. analysed the data; H.G. and L.A. led the writing of the manuscript. All authors contributed critically to the drafts and gave final approval for publication.

## DATA ACCESSIBILITY

Data have been deposited in the NERC Environmental Information Data Centre https://doi.org/10.5285/5321bc6e-be35-4ed3-9b56-25598d61ac8f (Griffiths et al., 2017).
