# Supplementary material for: Ants are the major agents of resource removal from tropical rainforests
Source: J Anim Ecol. 2017 Aug 8;87(1):293–300. doi: 10.1111/1365-2656.12728 (PMC6849798; doi:10.1111/1365-2656.12728)
Supplement: Supplementary file 1 [file JANE-87-293-s001.docx]

**Appendix 1 – Ant monitoring bait results**

Data from ant monitoring baits demonstrate that the ant suppression treatment significantly reduced the abundance of ants that arrived at monitoring cards (LRT = 8.93, df = 2, *P* = 0.01; determined by an ordered logistic regression using the clmm package in R; example syntax: m1 <- clmm(activity.score ~ Treatment + (1| Date) + (1| Plot), data = cards); Fig. S1). The mean activity score on bait cards in control plots was 2.5 (indicating a mean abundance of between 5 and 10 ants), whereas the mean score on the ant suppression plots was 0.24 (indicating a mean abundance of less than one ant). This demonstrates that the suppression treatment reduced the activity of ants on treatment plots by an average 90% compared to ant activity on control plots.


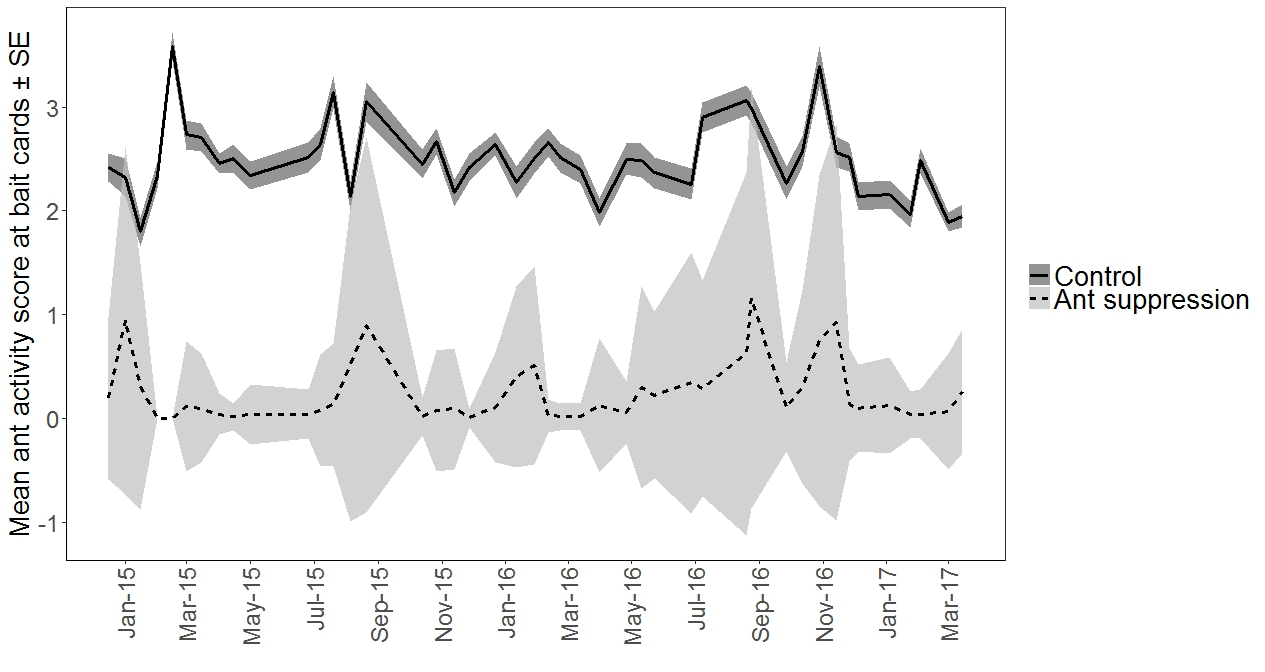


**Figure S1.1** Mean ant activity scores (± SE: shaded ribbons) at bait cards, assessed every two weeks for two years on control (solid line) and ant suppression plots (dashed line).
