# Supplementary material for: Ants are the major agents of resource removal from tropical rainforests
Source: J Anim Ecol. 2017 Aug 8;87(1):293–300. doi: 10.1111/1365-2656.12728 (PMC6849798; doi:10.1111/1365-2656.12728)
Supplement: Supplementary file 2 [file JANE-87-293-s002.docx]

**Appendix S2 – Details of cage treatment, baits, and plot spatial design**

**Cage treatments**

**
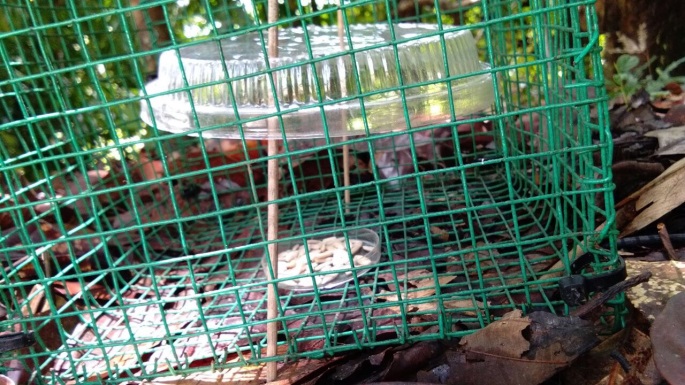

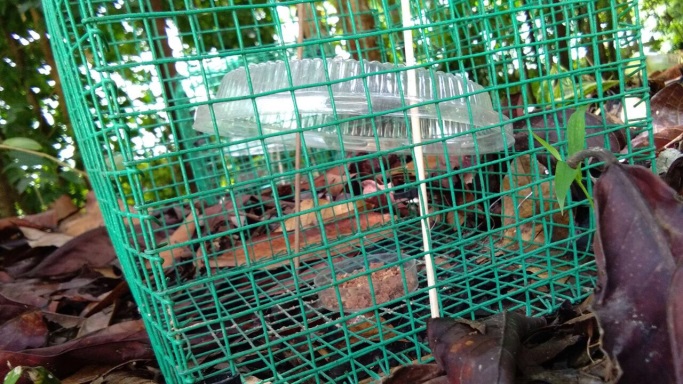
**

**Figure S2.1.** Caged bait stations, which restricted the access vertebrates to the food resources, containing seed (left photo) and carbohydrate bait (right photo)

**
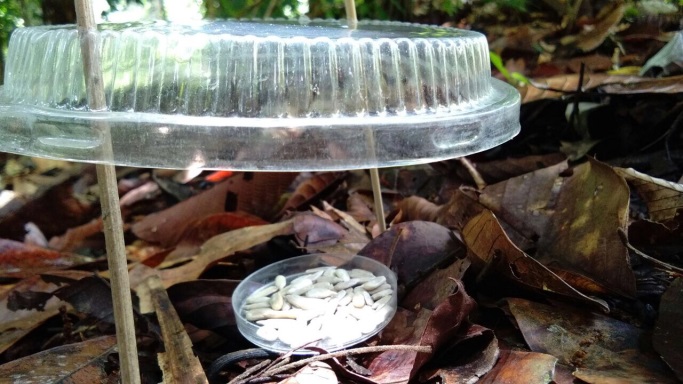

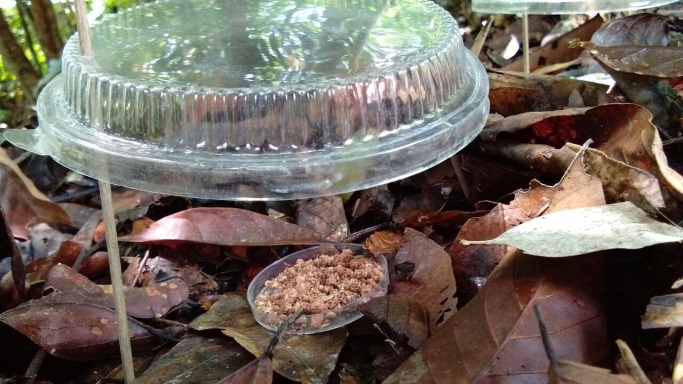
**

**Figure S2.2.** Open bait stations, allowing access by all foragers to the food resources, containing seed (left photo) and carbohydrate bait (right photo)

**Bait details**

**Carbohydrate bait:**

We used a wheat-based biscuit readily available in supermarket stores:

*Tigar Biskuat Choc*

Ingredients: Wheat flour, sugar, palm oil, cocoa powder, raising agents (ammonium bicarbonate), acid sodium pyrophosphate, liquid glucose, vitamins and minerals (A, B1, B2, B3, B5, B6, B12, D, E, Calcium carbonate, Magensium, Iron, Phosphorus, Iodine, Zinc), Milk powder, tapioca starch, salt, flavour.

**Protein bait:**

We used a dehydrated fish snack readily available in supermarket stores:

*Wanfa Snek Ifan*

Ingredients: Fresh fish meat, Wheat starch, Wheat flour, sugar, salt, chilli, flavourings

This product was chosen over fresh or tinned fish because it facilitated the quantification of dry mass removed by scavenger, whereas this would have been problematic with tinned or fresh fish.

**Spatial arrangement of bait stations on experimental plots**


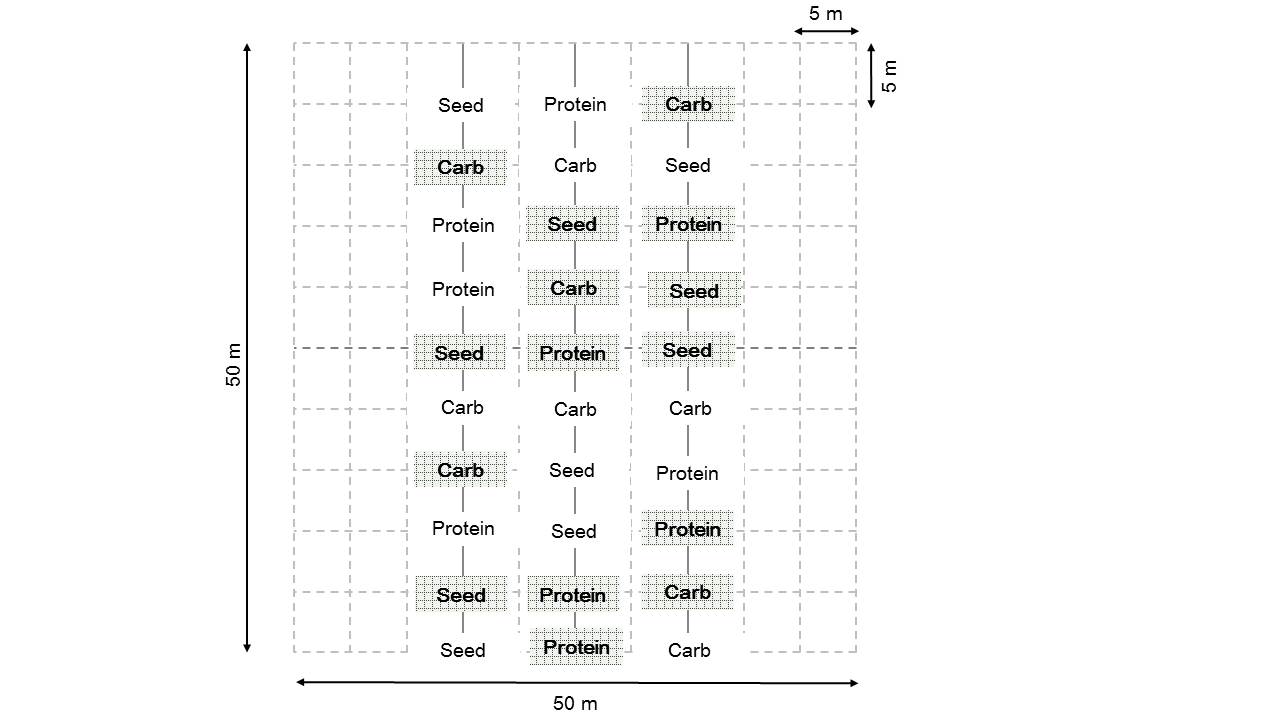


**Figure S2.3.** Bait x treatment spatial arrangement on an experimental plot. Each bait type was replicated five times per caged (hashed words)/open treatment and placed randomly on three, 50 m transects. Bait stations on the transects were 5 m apart and each transect was separated by 10 m. This resulted in n = 30 bait stations (5 bait replicates x 3 bait types x 2 cage treatments) for each of the eight plots (4 x ant suppression and 4 x control). This was repeated twice per plot totalling n = 480 bait stations for the experiment as a whole (60 baits x 4 plot replicates x 2 plot treatments [ant suppression/control])
