# Supplementary material for: Ants are the major agents of resource removal from tropical rainforests
Source: J Anim Ecol. 2017 Aug 8;87(1):293–300. doi: 10.1111/1365-2656.12728 (PMC6849798; doi:10.1111/1365-2656.12728)
Supplement: Supplementary file 3 [file JANE-87-293-s003.docx]

**Appendix S3 – Bait stations attacked by pigs**

We used a binomial glmer to assess whether plot treatment, cage treatment, or bait type influenced the likelihood that pigs disrupted bait stations. In total 103 bait stations (21.5%) were removed from the main analyses because of pig interference. No factor significantly affected the likelihood of pig attack (fig. 1).

**Figure S3.1.** Median proportion and interquartile range of bait stations (carbohydrate, protein, or seed) excluded from analyses experimental plots (ant suppression and control) because they were affected by pigs in the field. Caged baits are light blue boxes and baits open to all foragers are dark blue boxes. Outliers are displayed by black dots.
