# Supplementary material for: Ants are the major agents of resource removal from tropical rainforests
Source: J Anim Ecol. 2017 Aug 8;87(1):293–300. doi: 10.1111/1365-2656.12728 (PMC6849798; doi:10.1111/1365-2656.12728)
Supplement: Supplementary file 4 [file JANE-87-293-s004.docx]

**Appendix S4 – Differences in bait removal**

**Figure S4.1**. The mean (± SE) proportion of bait removed from ant suppression (Ant) and control plots (Control) from caged (light blue bars: vertebrate exclusion) and open bait stations (dark blue bars: open to all foragers).
